# Effectiveness of the Tobacco Tactics program in the Department of Veterans Affairs

**DOI:** 10.1186/1617-9625-12-S1-A12

**Published:** 2014-06-06

**Authors:** Sonia A Duffy, David Ronis, Carrie A Karvonen-Gutierrez, Lee A Ewing, Gregory W Dalack, Patricia M Smith, Timothy P Carmody, Thomas Hicks, Christopher Hermann, Pamela Reeves, Petra Flanagan

**Affiliations:** 1 Ann Arbor VA Center for Clinical Management Research, Health Services Research and Development, Ann Arbor, Michigan, 48105, USA; 2 School of Nursing, University of Michigan, Ann Arbor, Michigan, 48105, USA; 3 Department of Otolaryngology, University of Michigan, Ann Arbor, Michigan, 48105, USA; 4 Department of Psychiatry, University of Michigan, Ann Arbor, Michigan, 48105, USA; 5 Northern Ontario School of Medicine, Lakehead University, Sudbury, Ontario, P3E 2C6, Canada; 6 San Francisco VA Medical Center, San Francisco, California, 94121, USA; 7 Richard L. Roudebush VA Medical Center, Indianapolis, 46202, USA; 8 John D. Dingell VA Medical Center, Detroit, Michigan, 48201, USA; 9 VA Ann Arbor Healthcare System, Ann Arbor, Michigan 48105, USA

## Background

Smoking cessation interventions during hospitalization have been shown to be efficacious, yet are rarely incorporated into practice. The purpose of this study was to determine the effectiveness of the Tobacco Tactics program in three Veterans Affairs (VA) hospitals.

## Materials and methods

In this quasi-experimental pre- post- comparison effectiveness trial, inpatient nurses were educated to provide the Tobacco Tactics intervention in the Ann Arbor, MI and Detroit, MI VA hospitals, while the Indianapolis, IN VA hospital was the control site (N=1,070). The Tobacco Tactics nurse toolkit included: 1) one contact hour for training; 2) a PowerPoint presentation on behavioral and pharmaceutical interventions; 3) a pocket card “Helping Smokers Quit: A Guide for Clinicians”; 4) pharmaceutical and behavioral protocols; and 5) a computerized template for nurse documentation. The patient toolkit included: 1) a brochure; 2) a videotape “Smoking: Getting Ready to Quit;” 3) a Tobacco Tactics manual; 4) pharmaceuticals; 5) a 1-800-QUIT-NOW help line card; and 6) post-discharge telephone calls. Smoking patients were surveyed in the hospital and again six-months post-discharge. Urinary cotinine tests were used to verify six-month smoking status.

## Results

The average age was 55.3 years, most were male (94%) and not married (76%). After adjustment for the propensity of being assigned to treatment condition, there were significant improvements in 6-month quit rates in the pre- to post-intervention time periods in Ann Arbor (p=0.004) and Detroit (p<0.001) compared to the Indianapolis control site. The intervention was particularly effective in Detroit where pre-intervention quit rates were 4% compared to 13% post-intervention.

## Conclusions

This study showed that training staff nurses to integrate smoking cessation services into their routine care may increase quit rates. The Tobacco Tactics program, which meets the newly released (2011) Joint Commission standards that apply to all inpatient smokers, has the potential to significantly decrease smoking among patients admitted to VA hospitals.

